# Platelet Microparticles Enriched in miR-223 Reduce ICAM-1-Dependent Vascular Inflammation in Septic Conditions

**DOI:** 10.3389/fphys.2021.658524

**Published:** 2021-05-31

**Authors:** Bernadett Szilágyi, Zsolt Fejes, Ágnes Rusznyák, Ferenc Fenyvesi, Marianna Pócsi, Sándor Halmi, Zoltán Griger, Satya P. Kunapuli, János Kappelmayer, Béla Nagy

**Affiliations:** ^1^Department of Laboratory Medicine, Faculty of Medicine, University of Debrecen, Debrecen, Hungary; ^2^Kálmán Laki Doctoral School of Biomedical and Clinical Sciences, Faculty of Medicine, University of Debrecen, Debrecen, Hungary; ^3^Department of Pharmaceutical Technology, Faculty of Pharmacy, University of Debrecen, Debrecen, Hungary; ^4^Doctoral School of Pharmaceutical Sciences, University of Debrecen, Debrecen, Hungary; ^5^Faculty of Medicine, Institute of Internal Medicine, University of Debrecen, Debrecen, Hungary; ^6^Department of Physiology and Sol Sherry Thrombosis Center, Temple University School of Medicine, Philadelphia, PA, United States

**Keywords:** platelet, endothelial cell, microRNA, miR-223, sepsis, microparticle, ICAM-1

## Abstract

In the process of sepsis, activated platelets shed microvesicles containing microRNAs (miRNAs), which can be internalized by distinct recipient cells in circulation, consequently eliciting a potent capability to regulate their cellular functions in different diseases. In the present study, activated human platelets transferring miR-223 into endothelial cells via platelet-derived microparticles (PMPs) was investigated *in vitro* during septic conditions with a proposed mechanism involving in downregulation of the enhanced expression of intercellular adhesion molecule-1 (ICAM-1). The uptake of PMPs encasing miR-223 and the adhesion of peripheral blood mononuclear cells (PBMCs) on human coronary artery endothelial cells (HCAECs) were observed by immunofluorescence microscopy upon co-culture with PMPs isolated from sepsis or control plasma. The expression of miR-223-3p and its gene target *ICAM1* in HCAECs were quantified by RT-qPCR and ELISA after the cells were incubated with septic or control PMPs, whose levels were induced with thrombin-receptor activating peptide (TRAP). Leukocyte-depleted platelets (LDPs) from septic patients showed a decreased miR-223 level, while septic plasma and PMPs revealed an elevated miRNA level compared to control samples. Similarly, TRAP-activated LDPs demonstrated a reduced intracellular miR-223 expression, while increased levels in the supernatant and PMP isolates were observed vs. untreated samples. Furthermore, TNF-α alone resulted in decreased miR-223 and elevated *ICAM1* levels in HCAECs, while PMPs raised the miRNA level that was associated with downregulated *ICAM1* expression at both mRNA and protein levels under TNF-α treatment. Importantly, miR-223 was turned out not to be newly synthesized as shown in unchanged pre-miR-223 level, and mature miR-223 expression was also elevated in the presence of PMPs in HCAECs after transfection with Dicer1 siRNA. In addition, septic PMPs containing miR-223 decreased *ICAM1* with a reduction of PBMC binding to HCAECs. In conclusion, septic platelets released PMPs carrying functional miR-223 lower *ICAM1* expression in endothelial cells, which may be a protective role against excessive sepsis-induced vascular inflammation.

## Introduction

It has recently been established that platelet microRNAs (miRNAs) are involved in the fine-tune regulation of platelet translation (Landry et al., 2009; Kondkar et al., 2010; Szilágyi et al., 2020) and microvesicle generation (Lazar et al., 2020), consequently they undoubtedly affect platelet activation (Edelstein et al., 2013) and modulate response to anti-platelet therapy (Shi et al., 2013). Circulating miRNAs are present in the bloodstream in microvesicle-associated or protein-bound forms with distinct cell origin (Chen et al., 2012). Since activated platelets shed the largest amount of microvesicle containing functional miRNAs, the question has been raised whether these “novel vectors” can also play a role in intercellular signaling and messenger RNA (mRNA) regulation outside of platelets (Provost, 2017; Shu et al., 2019). Hence, this paracrine effect of miRNAs may have prospective diagnostic and therapeutic potentials in cardiovascular and malignant diseases (Provost, 2017).

In sepsis, platelets become activated and secrete various pro-inflammatory mediators, cytokines and components of proteolytic cascades, which target predominantly leukocytes and the endothelium (Dewitte et al., 2017). As a result, endothelial cells undergo physical changes causing the release of von Willebrand factor and enhanced expression of cell adhesion molecules including E-selectin, vascular cell adhesion molecule 1 and intercellular adhesion molecule-1 (ICAM-1) (Skibsted et al., 2013). Subsequently, platelets adhere to endothelial cells and vascular integrity is deleteriously affected. Recruited leukocytes in turn get activated and start to migrate to the site of inflammation via interacting with endothelial adhesion molecules (Dewitte et al., 2017), among which ICAM-1 is responsible for promoting slow rolling of leukocytes via binding to lymphocyte function-associated antigen 1 (LFA1) (Vestweber, 2015). Meanwhile, this receptor also participates in rolling, arrest, and transcellular diapedesis of leukocytes. ICAM-1 is highly expressed on the endothelial surface in the presence of activated platelets through an IL-1-dependent mechanism (Gawaz et al., 2000), but it becomes solubilized due to proteolytic cleavage (Vestweber, 2015). In sepsis, an elevated level of the circulating ICAM-1 can be utilized as a reliable biomarker that correlates with disease progression and clinical outcomes (de Pablo et al., 2013; Fang et al., 2018).

miR-223 is one of the most widely investigated miRNAs so far (Jeffries et al., 2019), which has been implicated in not limited to cell proliferation (Wu et al., 2012) and differentiation (Yuan et al., 2009), but it also possesses anti-inflammatory effects via inhibiting the NF-κB pathway and production of interleukins in endothelial cells (Dai et al., 2014) and downregulating the MAPK pathway in macrophages (Wang et al., 2016). Furthermore, miR-223 is highly expressed in platelets (Plé et al., 2012), which explains why its abnormal expression can contribute to endothelial disorders in cardiovascular diseases (Laffont et al., 2013) and diabetes mellitus (Pan et al., 2014). Also, according to sequence complementarity, miR-223 targets *ICAM1*, implying its involvement in endothelial response to inflammation; however, only two studies have focused on the effects of miR-223 on endothelial ICAM-1 expression (Tabet et al., 2014; Li et al., 2017), and no report is available on the regulatory role of this miRNA on *ICAM1* expression and its consequence among septic conditions. Therefore, in the present study, we investigated for the first time how platelet-derived microparticles (PMPs) enriched in miR-223 modulated *ICAM1* and decreased leukocyte-endothelium interaction in an *in vitro* model of sepsis.

The aims of this study are (i) to analyze the level of miR-223 in platelets, plasma samples and isolated PMPs from septic patients in comparison to control individuals, (ii) to observe the internalization of sepsis PMPs into endothelial cells, involving the transfer of miR-223, and (iii) to investigate the effects of PMPs containing miR-223 on ICAM-1 expression and leukocyte adhesion on endothelial cell cultures in the septic milieu.

## Materials and Methods

### Study Subjects, Blood Sample Preparation

In addition to routinely accessed whole blood and serum samples, venous blood samples were also obtained for platelet, plasma, and PMP miRNA analysis by atraumatic venipuncture into Vacutainer^®^ tubes containing 0.105 M sodium citrate (Becton Dickinson, San Jose, CA, United States) from septic patients (*n* = 13) after admission to the intensive care unit (ICU) of the Department of Internal Medicine and before any sepsis-specific treatment. Sepsis was diagnosed based on the criteria of the American College of Chest Physicians/Society of Critical Care Medicine Consensus, which defined systemic infection and 2 of the following: (a) temperature > 38°C or < 36°C; (b) heart rate > 90 beats/min; (c) respiratory rate > 20 breaths/min or PaCO_2_ < 32 mm Hg; (d) WBC count > 12,000/mm^3^, < 4,000/mm^3^, or > 10% bands (Bone et al., 1992). Sequential Organ Failure Assessment (SOFA) score was determined by the clinicians. Exclusion criteria for enrollment into this study included malignancy, autoimmune disease, pregnancy, severe thrombocytopenia, and acute myocardial infarction or acute ischemic stroke within 1 month. Age- and sex-matched control individuals (*n* = 13) were enrolled among volunteers or staff members from the Department of Laboratory Medicine. The main demographical and laboratory parameters of both study groups are presented in Supplementary Table 1.

Samples were processed within 60 min after collection by subsequent centrifugation at 170 *g* for 15 min at room temperature (RT) to obtain platelet-rich plasma (PRP). The upper layer of PRP was carefully transferred to a plastic tube to avoid any leukocyte contamination and was further centrifuged at 1,500 *g* for 15 min at RT to prepare platelet-poor plasma (PPP).

### Leukocyte-Depleted Platelet Preparation

Leukocyte-depleted platelet (LDP) samples were purified by anti-CD45-conjugated magnetic microbeads (Dynabeads,^®^ Invitrogen, Oslo, Norway) within 30 min of blood sampling, as we previously described from our laboratory (Fejes et al., 2017). Briefly, after the incubation of 2 mL PRP with beads for 30 min at RT, samples were inserted into a magnetic separator (Becton Dickinson) for 2 × 2 min, and the LDP sample was subsequently transferred into a fresh tube for additional centrifugation (1,500 *g*, 15 min, RT). The obtained platelet pellet was lysed with 1 mL TRI reagent (Molecular Research Center Inc., Cincinnati, OH, United States) and stored at −20°C until RNA isolation.

### Isolation of Platelet-Derived Microparticles

First, PPP was spun down at 13,000 *g* for 2 min at RT to get rid of platelet debris. PMPs were then harvested by two centrifugation steps at 16,100 *g* for 45 min at RT. Between the centrifugations, PMPs were washed with phosphate-buffered saline (PBS). The number of PMPs was determined by flow cytometry as described below.

### *In vitro* Activation of Platelets by TRAP for PMP Generation

For this experiment, LDP samples were prepared from 6 healthy donors. LDP specimens were treated with thrombin-receptor activating peptide (TRAP) (40 μM, Sigma-Aldrich, St Louis, MO, United States) at 37°C for 2 h under non-stirring conditions. Platelets were subsequently centrifuged (1,500 g, 15 min, RT), and the isolated platelet pellet was lysed with 1 mL TRI reagent, while 250 μL of platelet supernatant was lysed with 750 μL TRI reagent. Both sample lysates were stored at −20°C until RNA isolation.

### Culture of HCAECs

Human coronary artery endothelial cells (HCAECs, Cell Applications Inc., San Diego, CA, United States) were cultured in Meso Endo Cell Growth Medium (Cell Applications) at 37°C, 5% CO_2_ as we performed recently (Fejes et al., 2018). The cell count was set to 1 × 10^5^ per well in 6-well plates (Sigma-Aldrich). HCAECs were stimulated with PMPs isolated from septic or control plasma for 24 h. As a positive control group, HCAECs were treated with TNF-α (100 ng/mL, Gibco, Carlsbad, CA, United States) for 24 h. After treatment, cells were washed once with sterile Hanks’ Balanced Salt Solution (HBSS, Sigma-Aldrich), followed by lysis in 1 mL TRI reagent and stored at −20°C until RNA isolation.

### Total RNA Extraction

Total RNA including miRNAs was extracted from plasma, PMPs, LDPs, and HCAEC samples by TRI reagent according to the manufacturer’s recommendations. The purity and the concentration of separated RNA samples were measured by a NanoDrop spectrophotometer (Thermo Fisher Scientific, Wilmington, DE, United States). The obtained total RNA samples were stored at −80°C.

### miRNA Specific Stem-Loop RT-qPCR Analysis

The expression of extracellular and intracellular miR-223 was quantified by miRNA specific Universal ProbeLibrary (UPL)-probe based stem-loop RT-qPCR method (Fejes et al., 2017). The quantification technique included two steps: (1) miRNAs (input total RNA: 10 ng) were transcribed into cDNA via reverse transcription using miRNA-specific stem loop-RT primer (500 nM, Integrated DNA Technologies, Leuven, Belgium) and TaqMan MicroRNA Reverse Transcription Kit (Applied Biosystems, Foster City, CA, United States), and (2) miRNA quantification was performed by RT-qPCR using designed universal reverse primer (100 μM, Sigma-Aldrich), miRNA-specific forward primer (100 μM, Integrated DNA Technologies), and UPL probe #21 (10 μM, Roche Diagnostics, Mannheim, Germany) with Taq polymerase (5 U/μL, Thermo Fisher Scientific, Vilnius, Lithuania) and dNTPs (2.5 mM, Thermo Fisher Scientific). The reactions were preincubated at 95°C for 1 min, followed by 40 cycles of 95°C for 15 s and 60°C for 30 s. All the measurements were conducted in triplicate on a QuantStudio 12 K Flex qPCR instrument (Applied Biosystems). For normalization, the small-nucleolar RNU-43 was measured as a reference gene. Primers and qPCR assays were designed by the software developed by Czimmerer et al. (2013). Oligonucleotides of mature miR-223 that were used in this study are listed in Supplementary Table 2.

### RT-qPCR Analysis of mRNA and pre-miRNA

Complementary DNA (cDNA) synthesis was performed with High Capacity cDNA Reverse Transcription kit (Applied Biosystems) according to the manufacturer’s recommendation. The initial amount of RNA in LDP was 200 ng per reaction, while 1,000 ng per reaction was used in the HCAEC experiments. Quantitative PCR was performed on a QuantStudio 12 K Flex qPCR instrument with Light Cycler 480 SYBR Green I Master mix (Roche Diagnostics) and gene-specific primers (10 μM, Integrated DNA Technologies). The reactions were incubated at 95°C for 10 min, followed by 40 cycles of 95°C for 10 s and 60°C for 1 min. For normalization, the reference gene RPLP0 (36B4) was used. Sequences of the primers for *ICAM1* mRNA and pre-miR-223 are listed in Supplementary Table 2.

### Downregulation of *DICER1* Expression by siRNA Transfection in HCAEC Cells

Cells were plated at a density of 1 × 10^5^ per well for 24 h before transfection. *DICER1* expression in HCAEC cells was silenced by a specific siRNA (40 pmoL, ID: S23756, Invitrogen, Carlsbad, CA, United States) for 24 h as well as control samples with NEG01 siRNA (Silencer Select Negative Control No.1, Invitrogen, Carlsbad, CA, United States) according to the manufacturer’s recommendations, which is similar to our previous study (Szilágyi et al., 2020). Total RNA was then isolated, and the efficacy of transfection was monitored via the quantification of the Dicer1 siRNA level by TaqMan siRNA assay (ID: S23756_asy, Invitrogen, Carlsbad, CA, United States). The levels of miR-223, pre-miR-223 and DICER1 mRNA were measured by RT-qPCR thereafter.

### Measurement of Soluble ICAM-1 and TNF-α by ELISA

Serum samples kept at −80°C were analyzed by enzyme-linked immunosorbent assay (ELISA) measurements. The protein concentrations of ICAM-1 were determined in both sera (sepsis and control) and in the supernatants of treated HCAEC cultures by ELISA (Sigma-Aldrich) according to the manufacturer’s instructions. Additionally, TNF-α levels were measured in sepsis and control plasma samples by ELISA (R&D Systems, Minneapolis, MN, United States) for determining the applicability of TNF-α treatment in the cell culture model. Before the measurement, samples were centrifuged at 10,000 *g* for 1 min.

### Flow Cytometry

Surface P-selectin was analyzed by flow cytometry as we performed in a previous study (Csongrádi et al., 2011). Briefly, fixed platelets were incubated with saturating concentrations of fluorescein isothiocyanate (FITC)-conjugated monoclonal antibody to GPIX-receptor (CD42a) and phycoerythrin (PE)-labeled anti-P-selectin (CD62-PE, Becton Dickinson) for 20 min in the dark at RT to investigate the level of platelet activation. As a control for immunolabeling with anti-CD62 antibody, platelets were incubated with PE-coupled non-immune mouse IgG_1_ antibody. A total of 10,000 dual-color labeled platelet events were acquired on an FC-500 flow cytometer (Beckman Coulter, Pasadena, CA, United States). Results were expressed as the percentage of double-positive platelets.

The number of PMPs was quantified by a standardized method we set earlier (Csongrádi et al., 2011) with some minor modifications. PMP count was calculated based on the event count from the bead tube collected for the same time (60 s). PMPs were gated into a restricted area by forward scatter (FSC) and side scatter (SSC) parameters and then identified by Annexin V-FITC and CD41a-PeCy5 positivity.

### Investigation of PMP Uptake by Fluorescence Microscopy

Internalization of PMPs by endothelial cells was investigated in HCAECs that were cultured in 8-well plates (Millicell^®^ EZ slide, Millipore, Sigma-Aldrich) on sterile uncoated microscope slides at a density of 0.25 × 10^5^ cells per slide for 2 days. The cells were then treated with isolated septic or control PMPs or vehicle (PBS) for 24 h at 37°C. To exclude PMP incorporation into HCAECs via endocytosis and allow for exclusive focus on the effect of inhibited uptake of PMPs on miR-223, the incubation of HCAECs with PMP samples was performed on ice (at 4°C) similar to a former publication (Mizusako et al., 2015). Cells were then fixed with ice-cold methanol-acetone (50 v/v %) for 10 min. Non-specific antibody binding sites were blocked with FBS (Sigma-Aldrich) for 15 min. For fluorescence labeling HCAECs were incubated with mouse anti-human CD146-PE antibody (Becton Dickinson) for 1 h, while PMPs were labeled by mouse anti-CD42a-FITC (Becton Dickinson). Cell nuclei were dyed with 4′,6-diamidino-2-phenylindole (DAPI, 283 nM). Microscopic evaluation was performed using a Zeiss Axioscope.A1 fluorescent microscope (HBO 100 lamp) (Carl Zeiss Microimaging GmbH, Göttingen, Germany) (Szilágyi et al., 2020). DAPI: excitation 365 nm, emission BP 445/50 nm; fluorescein: excitation BP 470/40 nm, emission BP525/50 nm, PE: excitation BP 546/12 nm, emission 575–640 nm. Images were analyzed and the fluorescence intensity was quantified with ZEN 2012 v.1.1.0.0. software (Carl Zeiss Microscopy GmbH). The specificity of immunostaining was confirmed by incubating the cells with the secondary antibody only, and no background staining was found.

### Analysis of Leukocyte Adhesion to HCAECs by Fluorescence Microscopy

HCAECs were cultured on 8-well plates (Millicell^®^ EZ slide) at a density of 0.25 × 10^5^ per well for 2 days. HCAEC samples were then pretreated with isolated sepsis PMPs or PBS (control) for 24 h at 37°C. In addition, peripheral blood mononuclear cells (PBMCs) were separated from the buffy coat of control volunteers using Ficoll-Histopaque-1077 density gradient (Sigma-Aldrich). After washing with PBS, the number of PBMCs was set to 1 × 10^3^/mL using the same cell culture medium as HCAECs. Before co-culturing with HCAECs, PBMCs were stimulated with TNF-α (10 ng/mL) for 30 min and were then added to HCAECs for 1 h at 37°C. After incubation, the unattached PBMCs were washed out with HBSS three times and cells were fixed with ice-cold methanol-acetone (50 v/v %) for 10 min. Non-specific antibody binding sites were blocked with FBS (Sigma-Aldrich) for 10 min. HCAECs were stained with anti-CD146-PE antibody as described above, while PBMCs were labeled with anti-CD45-FITC antibody (Becton Dickinson). Cell nuclei were labeled with DAPI, and the samples were observed by Zeiss Axioscope.A1 fluorescent microscope (HBO 100 lamp) (Carl Zeiss Microimaging GmbH). DAPI: excitation 365 nm, emission BP 445/50 nm; fluorescein: excitation BP 470/40 nm, emission BP525/50 nm, PE: excitation BP 546/12 nm, emission 575–640 nm. The mean count of adhered PBMCs to HCAEC cells was determined in an equal number of microscopic fields (*n* = 5/condition).

### Other Laboratory Assays

White blood cell (WBC) and platelet (PLT) counts were determined by Advia 2120 Hematology System (Bayer Diagnostics, Tarrytown, NJ, United States). Serum C-reactive protein (CRP) and procalcitonin (PCT) levels were measured by electro-chemiluminescent immunoassay using a Cobas 8000 analyzer (Roche Diagnostics, Mannheim, Germany).

### Statistical Analysis

Kolmogorov–Smirnov test was used for evaluation of the normality of data. Results are expressed as mean ± SEM and median with interquartile range (IQR) as appropriate. To compare the data of two groups, unpaired *t*-test or Mann–Whitney *U*-test, and chi-squared test were applied. Statistical significance was defined when *p*-value was < 0.05. Statistical analyses were performed using GraphPad Prism software (version 6.01, La Jolla, CA, United States).

## Results

### miRNA Analysis Shows Decreased Expression of miR-223 in Activated Platelets Whereas Having Elevated Level in Plasma and Microparticles in Septic Patients

Thirteen patients suffering from sepsis were recruited into this study in parallel with 13 age- and sex-matched controls who did not suffer from any inflammatory disease at the time of enrollment. The main demographical and baseline parameters are summarized in Supplementary Table 1. As expected, WBC count raised, while platelet count reduced in sepsis subjects vs. controls (*P* < 0.01). Besides, serum C-reactive protein and procalcitonin levels were highly elevated in septic individuals based on their cut-off values (<4.6 mg/L and < 0.5 μg/L, respectively) indicating the development of severe inflammation. There was no difference in the administration of anti-platelet therapy between the two groups: there were 10 septic patients, who received aspirin/clopidogrel at admission, whereas 9 controls were administered on this treatment for the prevention of cardiovascular disease (Supplementary Table 1). Hence, the modulation of this therapeutic regimen on platelet activation and related miRNA levels could be excluded (Willeit et al., 2013).

**FIGURE 1:**
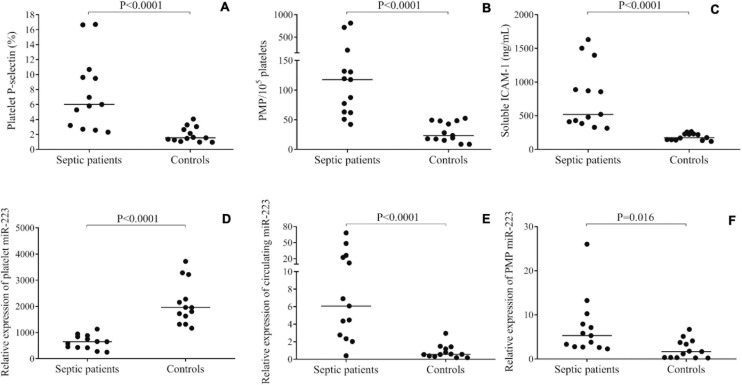
Evaluation of the level of platelet activation in septic patients vs. controls (*n* = 13/group). Surface P-selectin positivity **(A)**, the number of platelet-derived microparticles (PMPs) **(B)** and soluble intercellular adhesion molecule 1 (ICAM-1) concentration in serum samples **(C)** were significantly higher in sepsis vs. controls. Platelet miR-223 expression was decreased in sepsis **(D)**, however, plasma **(E)** and PMP samples **(F)** showed elevated miR-223 levels. Dots represent single results, and median values are depicted. Mann-Whitney *U*-test was performed for comparisons.

First, the level of platelet activation status was evaluated by surface P-selectin expression by flow cytometry (Kappelmayer et al., 2004). Similar to our recent sepsis study (Szilágyi et al., 2020), platelet P-selectin positivity was highly enhanced on septic vs. control platelets (6.1 [3.0–10.2] vs. 1.5 [1.1–2.8] %; *P* < 0.0001) (Figure 1A). We then quantified the number of PMPs in sepsis and control plasma samples using flow cytometry, and septic PMP count was significantly higher as compared to controls [117 (62–171) vs. 23 (16–48)/10^5^ platelets; *P* < 0.0001] (Figure 1B), which also evidenced the abnormal platelet activation in sepsis. In parallel, we analyzed soluble ICAM-1 level in sera to investigate the degree of endothelial cell activation at the early stage of sepsis (Skibsted et al., 2013), and the concentration was significantly augmented in sepsis [519 (397–1,142) vs. 174 (141–229) ng/mL; *P* < 0.0001] (Figure 1C). Based on these data, both platelet and endothelial cell activation were detectable in the septic subjects.

The expression of miR-223 was studied by RT-qPCR in three different types of sample: platelets, plasma, and isolated PMPs. We found that miR-223 expression was downregulated in sepsis platelets compared to controls (*P* < 0.0001) (Figure 1D), whereas circulating miR-223 level was elevated in plasma samples (*P* < 0.0001) (Figure 1E) and in PMPs (*P* < 0.016) (Figure 1F). These results imply that lower platelet miR-223 may be caused by the release of miRNA into the plasma environment via microvesicle generation due to triggered platelet activation under severe inflammatory conditions. Furthermore, we sub-grouped our sepsis subjects based on the severity and the outcome of the disease. Although there was a trend for lower platelet miR-223 in patients with septic shock (*n* = 8) vs. sepsis (*n* = 5) (*P* = 0.143) and in non-survivors (*n* = 10) vs. survivors (*n* = 3) (*P* = 0.076), no statistically significant difference was found probably due to the limited number of individuals (data not shown).

**FIGURE 2:**
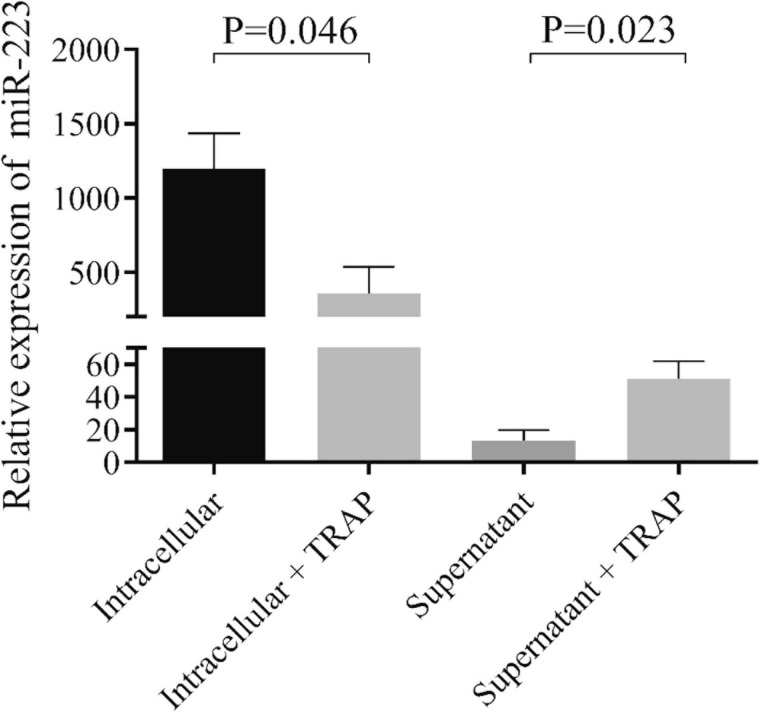
Analysis of miR-223 in platelets and their supernatants before and after TRAP activation *in vitro*. Isolated platelets were stimulated with thrombin-receptor activating peptide (TRAP, 40 μmol/L) for 2 h in non-stirring conditions. Platelets were then centrifuged, and platelet pellet and supernatant were processed for RNA extraction and miR-223 analysis. Platelets after stimulation (light gray column) showed decreased miR-223 levels compared to non-activated platelets (black column), while elevated miR-223 levels were detected in the supernatant with TRAP (gray column) (*n* = 7–8 samples/experiment). Mann-Whitney *U*-test was performed for comparisons.

To provide evidence on miRNA release from septic platelets, we performed *in vitro* experiments where control LDP samples were activated with TRAP for 2 h, followed by a separate examination of the supernatant as well as platelet pellet for miR-223. In TRAP-activated platelets, miR-223 expression significantly lowered in contrast to the untreated sample (*P* = 0.046), while increased miR-223 level was observed in the supernatant (*P* = 0.023) after treatment (Figure 2). RNU-43 was used as an internal control that showed a stable copy number in these samples above (data not shown). According to these results, we propose that induced platelet activation results in a substantial loss of platelet miRNAs and liberated miRNAs may mediate different cellular effects in sepsis.

### Internalization of Septic PMPs Is Enhanced Into Endothelial Cells

**FIGURE 3:**
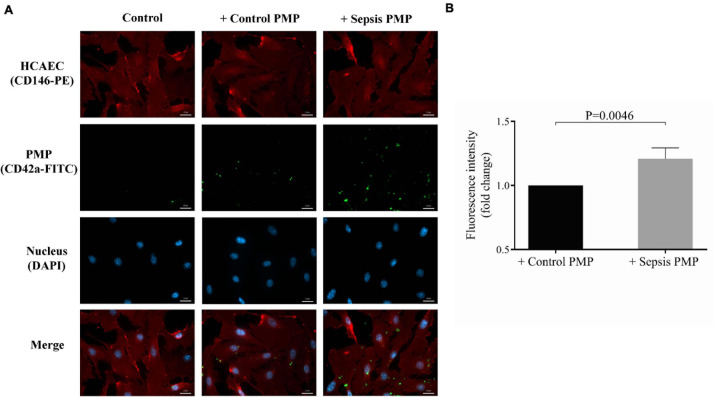
Immunostaining of the internalization of sepsis PMP by endothelial cells. Endothelial cells (HCAECs) were incubated with control and sepsis isolated platelet-derived microparticle (PMP) samples for 24 h at 37°C and HCAECs were then stained with anti-CD146-PE antibody (red) and PMPs were labeled with anti-CD42a-FITC antibody (green). The uptake of PMPs was investigated by fluorescence microscopy **(A)**. Blue: cell nuclei. Significantly higher fold change in fluorescence intensity (*P* = 0.0046) was detected in the cytosol of endothelial cells with septic PMPs compared to HCAECs with control PMPs **(B)**. Scale bar: 20 μm. Results are expressed as median with interquartile range (*n* = 5–6 cells/condition). Mann-Whitney *U*-test was performed for comparisons.

The endothelium is a key target of circulating microvesicles in cardiovascular and inflammatory diseases (Shu et al., 2019), therefore, we sought to investigate whether septic platelets transfer miRNAs into endothelial cells through microparticles to a higher extent than control counterparts. For this purpose, HCAEC cultures were co-cultured with isolated PMPs from sepsis or control individuals for 24 h at 37°C. Plasma samples that were added into the medium of endothelial cells contained a significantly higher level of PMPs than control samples [131 (76–279) vs. 48 (20–72)/10^5^ platelets, *P* = 0.020; *n* = 8/group] (data not shown). HCAECs were stained with anti-CD146-PE antibody against cell surface glycoprotein MUC18, and PMPs were labeled and localized via anti-CD42a-FITC (GPIX) positivity, as green-fluorescent staining was quantified intracellularly. Although PMPs were detected within HCAECs using either sepsis or control human samples, there was a higher level of green fluorescence in the case of HCAECs co-cultured with septic PMPs than controls (Figure 3A). Negative control HCAECs were maintained without PMP co-culture and showed no positivity. Also, we analyzed the fold change of fluorescence intensity, and there was a statistically significant difference (*P* = 0.0046) between sepsis and control samples based on the CD42a-FITC signal of endothelial cells (Figure 3B). These results demonstrate that PMPs can enter endothelium, and this cellular process is more pronounced under septic conditions vs. healthy conditions.

### PMPs Carrying Elevated miR-223 Level Repress ICAM1 Expression in HCAECs Under Septic Conditions

**FIGURE 4:**
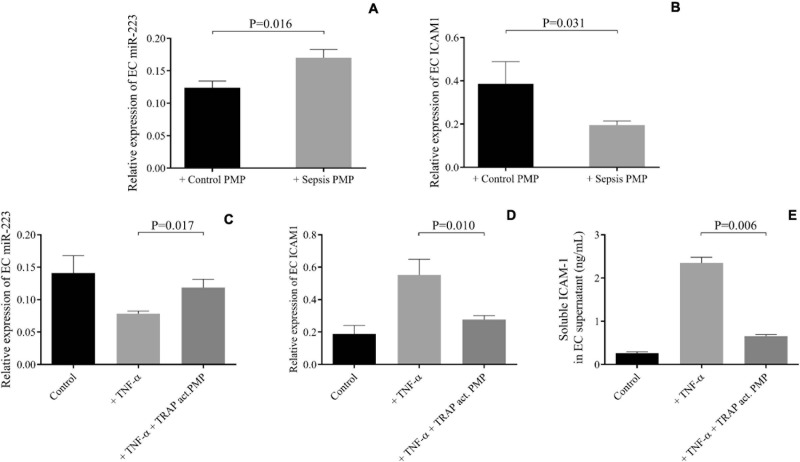
PMPs carrying elevated miR-223 repressed ICAM1 levels in HCAECs under septic conditions. Elevated miR-223 **(A)** and reduced intercellular adhesion molecule 1 (ICAM1) expression were detected in septic platelet-derived microparticle (PMP) stimulated endothelial cells (EC) after 24 h (gray columns) vs. HCAECs with control PMP (black columns) **(B)**. TNF-α alone (light gray columns) resulted in lower miR-223 and higher ICAM1 mRNA and protein levels. On the other hand, in the presence of thrombin-receptor activating peptide (TRAP) activated PMP samples (dark gray columns), upregulated miR-223 **(C)** and reduced ICAM1 mRNA **(D)** and protein levels **(E)** were found. Mean ± SEM was depicted (*n* = 7–8 samples/group). Mann-Whitney *U*-test or unpaired *t*-test was performed for comparisons.

Next, we wanted to observe the effect of sepsis PMPs on endothelial cell function via secreted miRNAs. HCAECs were first pretreated with isolated septic PMPs for 24 h, and elevated miR-223 expression was found (*P* = 0.016) compared to the control samples (Figure 4A), whereas ICAM1 mRNA level was reduced (*P* = 0.031) in the same set of samples (Figure 4B). To support these findings, HCAECs were incubated with PMPs generated from control platelets with TRAP stimulation *in vitro*. Since there was a significantly higher plasma TNF-α level in recruited sepsis patients in comparison to controls (28.6 ± 3.7 vs. 12.4 ± 1.1 pg/mL, *P* < 0.0001) (data not shown), the septic cellular conditions were mimicked using TNF-α on HCAECs. TNF-α pretreatment lowered miR-223 and upregulated ICAM1 mRNA level vs. control sample, while augmented miR-223 was detected (*P* = 0.017) with reduced *ICAM1* expression (*P* = 0.010) in the presence of TRAP-activated PMPs (Figures 4C,D). Soluble ICAM-1 in the supernatant of HCAECs was measured using ELISA and alterations were found in ICAM-1 concentrations similar to TNF-α upregulated ICAM-1 level, whereas cells with TNF-α and PMPs together showed significantly decreased protein concentration after 24 h (Figure 4E). These data underline that PMPs transporting miR-223 result in altered protein expression in endothelial cells under inflammatory circumstances.

### Inhibited Uptake of PMPs Into Endothelial Cells Prevents Change in miRNA Expression

To establish that modulated miR-223 expression was associated with the internalization of PMPs into endothelial cells, HCAECs were incubated for 6 h at 4°C with PMPs obtained from sepsis plasma samples vs. control cells without PMPs to prevent the endocytosis of microvesicles as reported earlier by others (Mizusako et al., 2015). As control, endothelial cells were co-cultured with or without PMPs at 37°C for the same time. HCAECs and PMPs were stained for fluorescence microscopy with the same set of antibodies described above. Immunostaining of PMPs revealed the uptake of septic PMPs into HCAECs was almost completely prevented on ice compared to cells incubated at 37°C (Supplementary Figures 1A,B). Interestingly, there was an increasing number of internalized PMPs into HCAECs from 1 to 24 h at 37°C (data not shown). During these blocking experiments at 4°C, HCAECs under PMP treatment for 6 h were selected, as endothelial cells underwent substantial morphological changes over 6 h of treatment that would have affected the analysis of PMP internalization. In addition, HCAECs were harvested, and total RNA was extracted for miR-223 analysis by RT-qPCR. We found that there was no change in miRNA expression in HCAEC samples maintained on ice (*P* = 0.969), while cells incubated with sepsis PMPs among normal cell culture conditions showed elevated miR-223 compared to the control sample without PMPs (*P* = 0.004) (Supplementary Figure 1C). These results confirm that increased miR-223 expression in HCAECs was directly caused by the endocytosis of PMPs carrying this miRNA.

### Altered miR-223 Expression Induced by PMPs Is Not the Consequence of miRNA Maturation or Increased Dicer1 Function in HCAECs

**FIGURE 5:**
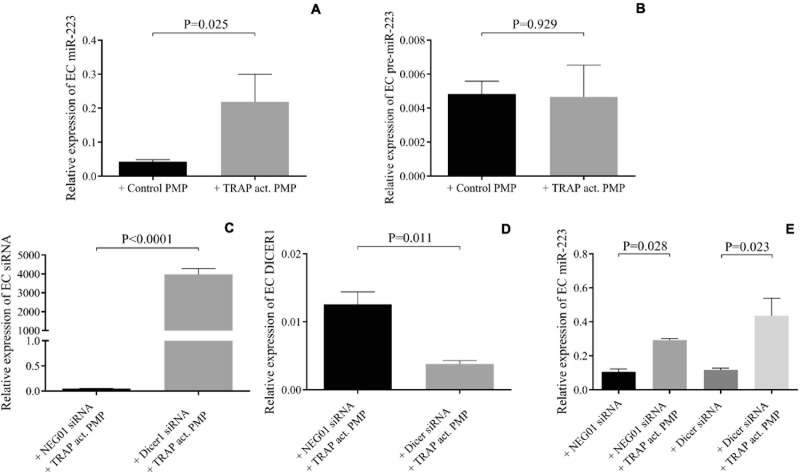
Altered miR-223 expression induced by PMPs was not the consequence of miRNA maturation or increased Dicer1 function in HCAECs. In the presence of thrombin-receptor activating peptide (TRAP) activated platelet-derived microparticle (PMP) (gray columns) increased miR-223 expression was detected in endothelial cells (EC) similar to sepsis PMP vs. HCAECs co-cultured with non-activated control PMP (black column) **(A)**. No difference was observed in pre-miR-223 level with or without TRAP-induced PMP excluding the effect of transcription **(B)**. Dicer1 specific siRNA was successfully transfected into endothelial cells **(C)** that resulted in reduced level of Dicer1 mRNA **(D)**. Despite reduced Dicer1 expression, miR-223 level was still significantly increased in the presence of TRAP-activated PMP in endothelial cells compared to samples without PMP **(E)**. Similar tendency was found in HCAECs with NEG-01 siRNA (black column). These results suggested that Dicer1 activity did not contribute to upregulated miR-223 caused by PMPs. Mean ± SEM was depicted (*n* = 4–5 samples/experiment). Mann-Whitney *U*-test or unpaired *t*-test was performed for comparisons.

We wanted to investigate whether other miRNA-related cellular events contributed to augmented miR-223 expression in HCAECs with septic PMPs to any extent. For this purpose, two *in vitro* experimental approaches were adopted: (i) analysis of the level of the precursor of miR-223 in the presence or absence of *in vitro* induced PMPs by TRAP, and (ii) silencing of the function of Dicer1 enzyme with Dicer1 siRNA pretreatment in HCAECs before the incubation with PMPs, in contrast to cells with NEG01 control siRNA. First, mature and pre-miR-223 levels were parallelly quantified by RT-qPCR in HCAECs with TRAP-activated PMPs vs. HCAECs co-cultured with non-activated control PMPs. The expression of the mature form of miR-223 was significantly (*P* = 0.025) enhanced by TRAP-activated PMPs, while no difference was observed in pre-miR-223 in these HCAEC samples (*P* = 0.929) (Figures 5A,B). Second, endothelial cells were transfected with Dicer1 siRNA or NEG01 siRNA in controls and isolated PMPs were added to the cells for 24 h. After total RNA extraction, the efficacy of transfection was confirmed via the quantification of Dicer1 siRNA level showing highly elevated expression (Figure 5C). This manipulation resulted in lowered DICER1 mRNA vs. control samples with NEG01 siRNA (Figure 5D). Importantly, similar to control cells in which Dicer1 function was not modulated with NEG01 siRNA, the mature miR-223 level was still significantly elevated (*P* = 0.023) in the presence of PMPs in HCAECs despite being transfected with Dicer1 siRNA (Figure 5E). In addition, reduced Dicer1 function did not cause any change in basal miR-223 level. Taken together, enhanced miR-223 level was attributed to the miRNA delivery by PMPs, and no transcription or induced Dicer1 activity contributed to the altered miR-223 expression of the septic endothelial cells.

### Adhesion of PBMCs to Endothelial Cells Is Reduced After Pretreatment With Septic PMPs

**FIGURE 6:**
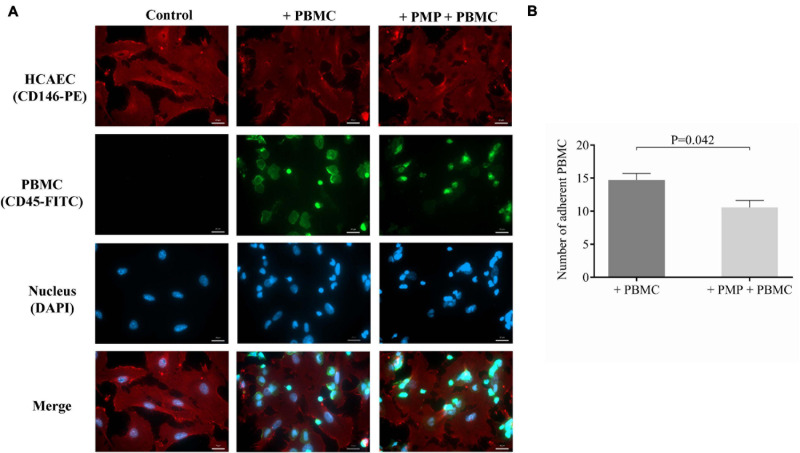
Investigation of reduced adhesion of PBMCs to endothelial cells after septic PMP treatment. HCAECs were seeded and pretreated with septic platelet-derived microparticles (PMPs) for 24 h. Cells were subsequently incubated with TNF-α-activated peripheral blood mononuclear cells (PBMCs) for 1 h. Endothelial cells were stained with anti-CD146-PE antibody (red) and PBMCs with anti-CD45-FITC antibody (green). The binding of PBMCs to the surface of endothelial cells was investigated by fluorescence microscopy **(A)**. Blue: cell nuclei. Less adherent PBMCs could be detected in PMP-pretreated HCAECs vs. non-treated cells **(B)**. Scale bar: 20 μm. Results are expressed as mean ± SEM. Mann-Whitney *U*-test was performed for comparisons. (*n* = 5–6 cells/condition).

Finally, a cell culture model was prepared to functionally investigate whether miR-223 via delivery with septic PMPs could reduce the attachment of leukocytes to the surface of endothelial cells due to the lower expression of ICAM-1. In this experiment, HCAECs were seeded and incubated with isolated and TNF-α activated PBMCs for 1 h with or without pretreatment with sepsis PMPs for 24 h. Negative control cells were maintained without PBMCs. HCAECs were stained with anti-CD146-PE antibody, while PBMCs were labeled and localized via CD45-FITC positivity. Using fluorescence microscopy, we evaluated the greenly fluorescence-stained leukocytes on the surface of HCAECs. We found that PBMCs were attached to the surface of endothelial cells and this phenomenon was reduced by 30% under the condition where PMPs were added to HCAECs (Figure 6A). The number of bound PBMCs without sepsis PMP pretreatment was 14 ± 2 per field vs. 10 ± 2 per field of PBMCs after preincubation with PMPs (Figure 6B). These data suggest that a lower level of ICAM-1 elicited by PMP-transferred miR-223 results in reduced attachment of leukocytes onto endothelium.

## Discussion

Sepsis represents a dysregulated systemic host response to microbial pathogens causing different vascular, metabolic, and cellular abnormalities (Singer et al., 2016). Sepsis-induced endothelial cell activation is associated with increased vascular inflammation and permeability as well as hemostasis imbalance with the abnormal microcirculatory flow (Hack and Zeerleder, 2001). The endothelium rapidly expresses the luminal adhesion molecules, such as ICAM-1 in response to various inflammatory triggers to promote leukocyte trafficking, while its soluble form then enters the circulation due to proteolytic cleavage (Vestweber, 2015).

Activated platelets not only play a key role in thrombus formation but also regulate pivotal functions of the cardiovascular system via releasing microvesicles and several bioactive substances (e.g., ADP, cytokines, RNA molecules, etc.) that affect the properties of individual cell types involved in atherosclerosis and sepsis (Badimon et al., 2016;Kerris et al., 2020). Despite lacking nuclei, platelets carry a large set of megakaryocyte-derived miRNAs (Plé et al., 2012). The functional role of certain platelet miRNAs has been recently uncovered in the platelet mRNA translation, such that miR-223 regulates the expression of P2Y12 receptor (Landry et al., 2009), VAMP8 expression is controlled by miR-96 (Kondkar et al., 2010), and miR-26b modulates *SELP* (P-selectin) expression in type 2 diabetes mellitus and sepsis (Fejes et al., 2017; Szilágyi et al., 2020). Platelet activation is accompanied by a secretion of miRNAs into the circulation. The liberated miRNAs can enter macrophages (Laffont et al., 2016) and endothelial cells (Laffont et al., 2013) and regulate different inflammatory responses and cell adhesion molecules (Zhong et al., 2018). Based on former findings, it has been revealed that HDL-transferred miR-223 targets and downregulates ICAM-1 expression in endothelial cells *in vitro* (Tabet et al., 2014), while platelet exosome enclosed miR-320b showed a substantial paracrine effect on endothelial ICAM-1 exposure in patients with myocardial infarction (Gidlöf et al., 2013). Accordingly, the cell-free miRNAs potentially fine-tune the effects of pro-inflammatory mediators in endothelial cells (Shu et al., 2019). To date, there is no available data on the role of platelet miR-223 in reducing endothelial ICAM-1 expression under septic conditions.

To perform *in vitro* experiments for this perspective, the samples of 13 sepsis patients and 13 controls were collected. It should be noted that there was no difference regarding anti-platelet therapy among the subjects, which excluded the impact of this medication on platelet activation-related miRNA expression as described earlier (Willeit et al., 2013). Enhanced platelet activation was demonstrated via high surface P-selectin exposure similar to our recent sepsis study (Szilágyi et al., 2020), and increased PMP levels in sepsis as compared to controls (Kappelmayer et al., 2013). Soluble ICAM-1 level was also augmented in sera indicating endothelial cell activation at the early stage of sepsis (Skibsted et al., 2013). Afterward, the expression of miR-223 was studied in platelets, plasma, and isolated PMPs of recruited individuals by RT-qPCR. miR-223 expression was downregulated in sepsis platelets, and to the contrary, circulating miR-223 level was elevated in both septic plasma samples and isolated PMPs vs. control specimens. Concordantly, recent sepsis studies reported that platelet miR-223 was lower than normal (Szilágyi et al., 2020), whereas circulating miR-223 was upregulated in septic subjects with pneumonia (Zhang et al., 2019) and normal individuals after induced endotoxemia (Braza-Boïls et al., 2020).

We supposed that the lower platelet miR-223 may be caused by the release of miRNA into the plasma environment via PMPs under these severe inflammatory conditions. Hence, we stimulated LDP samples with a high concentration of TRAP mimicking the increased level and the effect of thrombin generation in sepsis (Tóth et al., 2017), and the supernatant with platelet pellet was examined for miR-223. In TRAP-activated platelets, miR-223 expression was significantly lowered in contrast to the untreated sample, while increased miR-223 level was observed in the supernatant after treatment. Accordingly, the result translates into induced platelet activation, in turn resulting in a substantial transfer of miRNA within PMPs into the circulation. In agreement with our data, it has been reported that thrombin-induced platelet exosomes were enriched in miR-223 vs. untreated samples (Li et al., 2017). Also, thrombin activation caused a massive elevation in cel-miR-39 expression in the supernatant of platelets and a corresponding decrease in intracellular cel-miR-39 (Gidlöf et al., 2013).

The endothelium is a key target of circulating microvesicles in cardiovascular and inflammatory diseases (Shu et al., 2019), which attracted our attention to the investigation of septic platelets transferring miRNAs into the endothelial cells through microparticles for the first time. HCAEC cultures were co-cultured with isolated PMPs from sepsis or control individuals. Although both septic and control PMPs were detected within HCAECs, there was a larger uptake of septic PMPs into endothelial cells than controls after 24 h. Previous studies described the internalization of normal PMPs or exosomes into human umbilical vein endothelial cells (HUVECs) (Laffont et al., 2013; Li et al., 2017). In our current study, we reported the enhanced delivery of septic PMPs to the endothelium that could be inhibited at 4°C. This approach was previously applied by other researchers where the aim was to prevent cellular uptake by endocytosis (Jiao et al., 2009; Mizusako et al., 2015).

Next, we wanted to observe the effect of septic PMPs on endothelial cell function via secreted miRNAs. HCAECs were pretreated with septic PMPs showing elevated miR-223 expression, while ICAM1 mRNA level was suppressed. In parallel, TNF-α pretreated HCAECs were incubated with PMPs generated by TRAP stimulation *in vitro.* In contrast to activated HCAECs exhibiting lower miR-223 and upregulated ICAM1 mRNA levels, augmented miR-223 was detected with reduced *ICAM1* expression in the presence of induced PMPs. Importantly, the quantity of soluble ICAM-1 in the supernatant of endothelial cells was increased by TNF-α, while treatment with TNF-α and PMPs together resulted in significantly decreased protein concentration after 24 h. Additionally, there was no change in miRNA expression in HCAECs maintained on ice, while those among normal cell culture conditions at 37°C showed elevated miR-223 compared to the control sample without PMPs. Collectively, these results confirm that the endocytosis of PMPs carrying miR-223 increases the expression of this miRNA in HCAECs. Furthermore, PMP-transferred miR-223 is likely responsible to alter ICAM-1 protein expression in endothelial cells under inflammatory circumstances. Of note, the effect of PMP-derived miR-223 was not further confirmed via using specific miRNA inhibitor. Hence, other targets may be also affected, and additional miRNAs or mediators carried by PMPs cannot be excluded to contribute to altered ICAM-1 levels under these septic conditions.

Since the largest pool of microvesicles is derived from platelets, a substantial ratio of cell-free miRNAs is transferred from activated platelets into other cell types and interfere with gene expression (Águila et al., 2020). Based on former results, platelet-derived miR-223 regulated *EFNA1* and *FBXW7* gene expression at mRNA level in HUVECs (Laffont et al., 2013), while others reported the inhibitory role of platelet miR-320b on endothelial ICAM-1 expression after myocardial infarction (Gidlöf et al., 2013). It should be emphasized that *ICAM1* is not merely regulated by miR-223, but other miRNAs, such as miR-141 repressed ICAM-1 expression on the endothelium to attenuate myocardial ischemia-reperfusion injury (Liu et al., 2015). On the other hand, miR-223 also targets the expression of other proteins, e.g., insulin-like growth factor 1 receptor on vascular smooth muscle cells (Su et al., 2020).

To further elucidate the molecular mechanism of the miRNA expression, whether miRNA transcription and/or increased function of Dicer1 contributed to augmented miR-223 expression in HCAECs with septic PMPs was measured. To achieve this goal, we analyzed the level of the precursor of miR-223 in the presence or absence of *in vitro*-induced PMPs by TRAP. Methodologically, the expression with Dicer1 was silenced with siRNA pretreatment in HCAECs before the incubation with PMPs in contrast to cells with NEG01 control siRNA as we recently performed in MEG-01 cells (Szilágyi et al., 2020). First, the mature form of miR-223 was significantly enhanced by PMPs, while no difference was observed in pre-miR-223 in HCAECs as found by Tabet et al. (2014). Second, even in the presence of successful Dicer1 downregulation mature miR-223 level was still significantly elevated in the presence of PMPs in HCAECs despite being transfected with Dicer1 siRNA. Taken together, enhanced miR-223 level was a consequence of the miRNA delivered by PMPs, also suggesting that no transcription or induced Dicer1 activity contributed to the altered miR-223 expression of these septic endothelial cells. Of note, Dicer1 is critical in the modulation of gene expression and function in human endothelial cells via maintaining and regulating endogenous miRNAs. Dicer knockdown resulted in increased nitrogen-oxide release with reduced proliferation and impaired cord formation, but miR-223 and ICAM-1 expression were not affected (Suárez et al., 2007). In our current study, we did not find any difference either after Dicer1 manipulation in miR-223, while PMPs raised its level regardless of Dicer1 function.

Finally, to deepen understanding of the molecular effect of miR-223 on leukocyte interaction, a cell culture model was constructed, which enabled the functional investigation of whether septic PMPs with the delivery of miR-223 could attenuate the attachment of leukocytes to the surface of endothelial cells due to lower expression of ICAM-1. PBMCs bound to the surface of endothelial cells, which was reduced by 30% when PMPs were added to HCAECs as pretreatment. These data suggest that a lower level of ICAM-1 caused by PMPs with miR-223 results in reduced attachment of leukocytes to the endothelium. Due to the involvement of other receptors in leukocyte-endothelium interaction (Vestweber, 2015), we did not expect a higher extent of inhibition in the case of this cell culture model experiment.

There are some limitations of this study. First, we enrolled a limited number of sepsis subjects to provide human samples for platelet, plasma and PMP isolates for the *in vitro* experiments, however, significant differences were observed even at this low number of cases. Second, we directly analyzed the modulatory effect of TNF-α on endothelial cells concerning MP internalization in the early phase of sepsis, however, several other pro-inflammatory and infection-related mediators/cytokines (e.g., lipopolysaccharide) are also released into the circulation at a higher quantity, which may also influence endothelial functions. Third, we did not further investigate whether a selective redistribution of miR-223 takes place from LDPs to PMPs in the septic milieu, which brought us a further question if this event may also contribute to the lower expression of several platelet miRNAs along with the abnormal miRNA content of megakaryocytes in sepsis (Szilágyi et al., 2020). Hence, further studies are required to examine the molecular mechanism of miRNA transfer from activated platelets into different recipient cells via microvesicles in sepsis or other disorders.

## Conclusion

**FIGURE 7:**
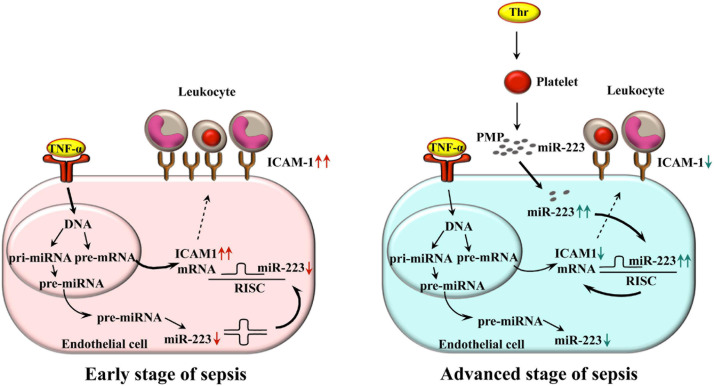
Schematic figure about the working model on the delivery of miR-223 by PMPs to endothelial cells to downregulate ICAM-1 expression and leukocyte adhesion in septic conditions. Inflammation triggered endothelial cells express elevated level of ICAM-1 on their surface in the early stage of sepsis (left panel). During the propagation of the disease, thrombin-stimulated platelets release a large amount of PMPs transferring miR-223 to different recipient cells. After the uptake by the endothelium, inflammation-induced ICAM-1 expression is reduced, which also limits leukocyte adhesion, acting as a protection against excessive sepsis-related vascular inflammation (right panel). Bold arrows depict the major signaling pathway in each stage of sepsis that affects ICAM-1 expression. ICAM-1, intercellular adhesion molecule-1; Thr, thrombin; TNF-α, tumor necrosis factor alpha; PMP, platelet microparticle; RISC, RNA-induced silencing complex.

The novelty of this work is the determination and elucidation of the functional effects of internalized septic PMPs enriched in miR-223 into endothelial cells on ICAM-1 expression and associated leukocyte adhesion under septic conditions. Inflammation-triggered endothelial cells express an elevated level of ICAM-1 on their surface in the early stage of sepsis. Based on our current data, during the propagation of the disease, thrombin-stimulated platelets release a large number of PMPs transferring miR-223 to endothelial cells. After the uptake by endothelium, inflammation-induced ICAM-1 expression is reduced, which also limits leukocyte adhesion acting as a protection against excessive sepsis-related vascular inflammation (Figure 7). The explication of this process may lead to the development of new therapeutic modalities to improve the circulatory function in sepsis.

## Data Availability Statement

The original contributions presented in the study are included in the article/Supplementary Material, further inquiries can be directed to the corresponding author/s.

## Ethics Statement

The study was approved by the Ethics Committee of the University of Debrecen (permit number: 4780-2017) in accordance with the Declaration of Helsinki.

## Author Contributions

BS, ZF, and MP performed the majority of experiments, analyzed data, and drafted the manuscript. ÁR and FF performed part of experiments and the data analyses. SH and ZG recruited study individuals and provided the patient samples. JK and SK performed the data analysis and reviewed the manuscript. BN directed all aspects of the experiments, and the data analysis manuscript editing and review. All authors contributed to the article and approved the submitted version.

## Conflict of Interest

The authors declare that the research was conducted in the absence of any commercial or financial relationships that could be construed as a potential conflict of interest.

## Supplementary Material

Supplementary Figure 1 Inhibited endocytosis of PMPs into endothelial cells prevents change in miRNA expression. HCAECs were incubated with platelet-derived microparticles (PMPs) obtained from sepsis plasma samples at 4°C for 6 h vs. control cells without PMPs to prevent the endocytosis of microvesicles. In contrast, endothelial cells were co-cultured with or without PMPs at 37°C for the same time. HCAECs were stained with anti-CD146-PE antibody (red) and PMPs were labeled with anti-CD42a-FITC antibody (green). The uptake of PMPs investigated by fluorescence microscopy was almost completely abolished in HCAECs on ice vs. cells among normal conditions **(A,B)**. Blue: cell nuclei. HCAECs were harvested and were analyzed for miR-223 by RT-qPCR. There was no change in miRNA expression in samples maintained at 4°C, while cells among normal cell culture conditions (at 37°C) showed elevated miR-223 compared to control samples without PMPs **(C)**. Scale bar: 20 μm. Results are expressed as mean ± SEM. (*n* = 4–5 cells or samples/condition). Mann-Whitney *U*-test was performed for comparisons.

Supplementary Table 1 The main demographical and laboratory parameters of both study groups. Septic patients (*n* = 13) were enrolled after admission to the intensive care unit (ICU) of the Department of Internal Medicine and sample collection was conducted prior to any sepsis-specific treatment. WBC count raised, while platelet count reduced in sepsis subjects vs. controls (*P* < 0.01). In addition, serum C-reactive protein (CRP) and procalcitonin (PCT) levels were highly elevated in septic individuals based on their cut-off values (<4.6 mg/L and <0.5 μg/L, respectively) indicating the development of severe inflammation. Sepsis was diagnosed based on the criteria of the American College of Chest Physicians/Society of Critical Care Medicine Consensus. Sequential Organ Failure Assessment (SOFA) score was determined by the clinicians. Age- and sex-matched control individuals (*n* = 13) were enrolled among volunteers or staff members from the Department of Laboratory Medicine. Results are expressed as mean ± SEM or median with interquartile range (IQR), as appropriate. Unpaired *t*-test or Mann–Whitney *U*-test, and chi-squared test was performed for comparisons.

Supplementary Table 2 Sequences of primers that were used in RT-qPCR analysis. In case of stem-loop reverse transcription primers, the miRNA specific sequences are highlighted in red. Universal ProbeLibrary probe #21 (5′—TGGCTCTG—3′) was used for miRNA measurements.

## Abbreviations

cDNA: complementary DNA
EC: endothelial cell
ELISA: enzyme-linked immunosorbent assay
FBS: fetal bovine serum
FITC: fluorescein isothiocyanate
FSC: forward scatter
HBSS: Hanks’ Balanced Salt Solution
HCAEC: human coronary artery endothelial cell
HDL: high density lipoprotein
ICAM-1: intercellular adhesion molecule-1
ICU: intensive care unit
LDP: leukocyte-depleted platelet
mRNA: messenger RNA
miRNA: microRNA
NF- κ B: nuclear factor kappa B
PBMC: peripheral blood mononuclear cell
PBS: phosphate-buffered saline
PE: phycoerythrin
PMP: platelet-derived microparticle
PPP: platelet poor plasma
PRP: platelet-rich plasma
RT: room temperature
RT-qPCR: real-time quantitative polymerase chain reaction
siRNA: small interfering RNA
SOFA score: Sequential Organ Failure Assessment score
SSC: side scatter
TNF- α: tumor necrosis factor alpha
TRAP: thrombin-receptor activating peptide
UPL-probe: Universal Probe Library
WBC: white blood cell.
