# Supplementary figures and images for: Platelet Microparticles Enriched in miR-223 Reduce ICAM-1-Dependent Vascular Inflammation in Septic Conditions
Source: Front Physiol. 2021 May 31;12:658524. doi: 10.3389/fphys.2021.658524 (PMC8201999; doi:10.3389/fphys.2021.658524)

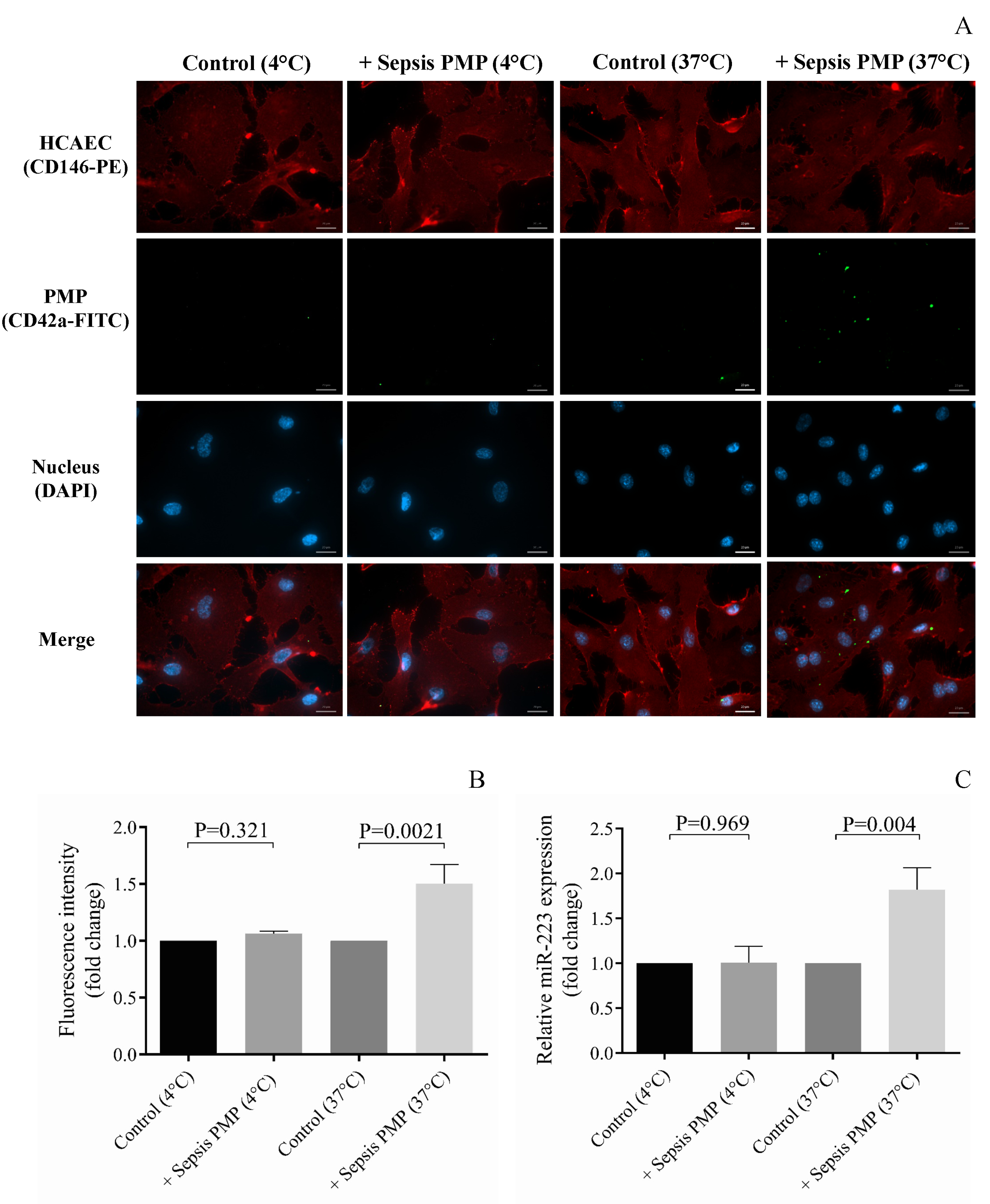

Supplement: Supplementary Figure 1 — Inhibited endocytosis of PMPs into endothelial cells prevents change in miRNA expression. HCAECs were incubated with platelet-derived microparticles (PMPs) obtained from sepsis plasma samples at 4°C for 6 h vs. control cells without PMPs to prevent the endocytosis of microvesicles. In contrast, endothelial cells were co-cultured with or without PMPs at 37°C for the same time. HCAECs were stained with anti-CD146-PE antibody (red) and PMPs were labeled with anti-CD42a-FITC antibody (green). The uptake of PMPs investigated by fluorescence microscopy was almost completely abolished in HCAECs on ice vs. cells among normal conditions (A,B). Blue: cell nuclei. HCAECs were harvested and were analyzed for miR-223 by RT-qPCR. There was no change in miRNA expression in samples maintained at 4°C, while cells among normal cell culture conditions (at 37°C) showed elevated miR-223 compared to control samples without PMPs (C). Scale bar: 20 μm. Results are expressed as mean ± SEM. (n = 4–5 cells or samples/condition). Mann-Whitney U-test was performed for comparisons. [file Image_1.TIF]
